# *BMC Medical Research Methodology* reviewer acknowledgement 2014

**DOI:** 10.1186/s12874-015-0003-4

**Published:** 2015-04-15

**Authors:** Giulia Mangiameli

**Affiliations:** BioMed Central, Floor 6, 236 Gray’s Inn Road, London, WC1X 8HB UK

## Abstract

The editors of BMC Medical Research Methodology would like to thank all our reviewers who have contributed to the journal in Volume 14 (2014).

**Erin Abner**

USA

**Esharenana E. Adomi**

Nigeria

**Julie Agel**

USA

**Arthur Allignol**

Germany

**Margaret Allman-Farinelli**

Australia

**Heather Allore**

USA

**Ming-Wen An**

USA

**Viola Angelini**

Netherlands

**Kristopher Attwood**

USA

**Matthew Baldwin**

USA

**Heejung Bang**

USA

**Adrian Barnett**

Australia

**Huiman Barnhart**

USA

**Eric Barrette**

USA

**Jonathan Bartlett**

UK

**Heather Battles**

New Zealand

**Mickael Bech**

Denmark

**Melanie Bell**

USA

**Sally Bell-Syer**

UK

**Eran Bendavid**

USA

**Ralf Bender**

Germany

**Vance Berger**

USA

**Nancy Berkman**

USA

**Regina Bernal**

Brazil

**Boris Bershadsky**

USA

**Sahul Bharti**

India

**Akbar Biglarian**

Iran

**Evdokia Billis**

Greece

**Harald Binder**

Germany

**Ingrid Binswanger**

USA

**Ann Blanc**

USA

**Paul Blanche**

Denmark

**Emily Blood**

USA

**Ben Bolker**

Canada

**Lyndal Bond**

Australia

**Billie Bonevski**

Australia

**Isabelle Boutron**

France

**Ulisses Braga-Neto**

USA

**Ruta Brazauskas**

USA

**Ann Bi Bremander**

Sweden

**Frank Bretz**

Switzerland

**Britton Brewer**

USA

**Camille Brisset**

Canada

**Nicky Britten**

UK

**Maarten Buis**

Germany

**Helen Burchett**

UK

**Alex Burdorf**

Netherlands

**Paul-Christian Bürkner**

Germany

**Elaine Burns**

UK

**Steve Buyske**

USA

**Julio Cabrero-Garcia**

Spain

**Chunyan Cai**

USA

**Lisa Calderwood**

UK

**Jerrilyn Cambron**

USA

**John Cape**

UK

**John Carlisle**

UK

**Bernie Carter**

UK

**Kristie Carter**

New Zealand

**Susan Cassels**

USA

**Ya-Mei Chang**

Taiwan

**Anne Chao**

Taiwan

**Yiyi Chen**

USA

**Chyong-Mei Chen**

Taiwan

**Hua Yun Chen**

USA

**Maggie Chen**

Canada

**Shuo Chen**

USA

**Xiwei Chen**

USA

**Zhuo Chen**

USA

**Sylvie Chevret**

France

**Chin-Tsang Chiang**

Taiwan

**Sangbum Choi**

USA

**Miew Keen Choong**

Australia

**Jody Ciolino**

USA

**Jan Clement**

USA

**Nancy Cook**

USA

**Giuliana Cortese**

Italy

**Susan Cox**

Canada

**Colin Crooks**

UK

**Gillian Currie**

UK

**Eleonora Dal Grande**

Australia

**Abhik Das**

USA

**Philippa Davies**

UK

**Z. John Daye**

USA

**Annemarie De Greeff**

UK

**Joris De Groot**

Netherlands

**Heather Dean**

Canada

**Thomas Debray**

Netherlands

**Sofia Dias**

UK

**Markus K. Diener**

Germany

**Philipp Doebler**

Germany

**Sarah Donegan**

UK

**Amylou Dueck**

USA

**Steven Duffy**

UK

**Stephanie Eckman**

Germany

**Solvig Ekblad**

Sweden

**Andrew Elders**

UK

**Paul B. English**

USA

**Diane Fairclough**

USA

**Libero Fatti**

South Africa

**Todd Favorite**

USA

**Lambert Felix**

UK

**Georg Ferber**

Switzerland

**Mallorie Fiero**

USA

**Krista Fischer**

Estonia

**Hans Flaatten**

Norway

**Benjamin Fletcher**

UK

**Tamsin Ford**

UK

**Jan Friedrich**

Canada

**Alex Fu**

USA

**Carmen Fuentelsaz-Gallego**

Spain

**James Gallagher**

UK

**Margaret Gamalo**

USA

**Claudia Geue**

UK

**Debashis Ghosh**

USA

**Tracy Glass**

Switzerland

**Roberta Goldman**

USA

**Mithat Gonen**

USA

**Adam L Gordon**

UK

**Jessica Gorman**

USA

**Inigo Gorostiza**

Spain

**Sue Grady**

USA

**Emily Griffith**

USA

**Catharina Groothuis-Oudshoorn**

Netherlands

**Martin Gulliford**

UK

**Kurinchi Gurusamy**

UK

**Chad Gwaltney**

USA

**Melissa Habel**

USA

**Alix Hall**

Australia

**Gill Hall**

New Zealand

**Bernhard Haller**

Germany

**Michele Hamm**

Canada

**Sebastien Haneuse**

USA

**Anthony Hanley**

Canada

**Frank Harrell**

USA

**Katie Harron**

UK

**Yulei He**

USA

**Weili He**

USA

**Dick Heasman**

UK

**Karla Hemming**

UK

**Anne Hickey**

Ireland

**Ralf-Dieter Hilgers**

Germany

**Niels Henrik Hjollund**

Denmark

**Zoe Hoare**

UK

**Rebecca Holman**

Netherlands

**Jeremy Howick**

UK

**Annika Hoyer**

Germany

**Iztok Hozo**

USA

**Asbjørn Hróbjartsson**

Denmark

**Chiu-Hsieh (Paul) Hsu**

USA

**Bo Hu**

USA

**Yueqin Hu**

USA

**Rebecca Hubbard**

USA

**Carmel Hughes**

UK

**Matthew Hutmacher**

USA

**Thao Huynh**

Canada

**Grace Irimu**

Kenya

**Dan Jackson**

UK

**Zeb Jamrozik**

Australia

**Zhenyu Jia**

USA

**Zhen Jiang**

USA

**Benjamin Johns**

USA

**Grace Johnston**

Canada

**Shahab Jolani**

Netherlands

**Galen Joseph**

USA

**Janet Jull**

Canada

**Marie-Laure Kaiser**

Switzerland

**Zeynal Karaca**

USA

**Ghassan Karam**

Switzerland

**Juha Karvanen**

Finland

**Benjamin Kasenda**

Switzerland

**Monika Kastner**

Canada

**Jacob Kean**

USA

**Heather Keller**

Canada

**Joanna Kesten**

UK

**Diba Khan**

USA

**Jane Kim**

USA

**Jonathan Kimmelman**

Canada

**Shiho Kino**

Japan

**Maria Kleinstaeuber**

Germany

**Stefanie Klug**

Germany

**Guido Knapp**

Germany

**Masha Kocherginsky**

USA

**Ruwanthi Kolamunnage Dona**

UK

**Maiying Kong**

USA

**Jochem König**

Germany

**Daniel Kotz**

Netherlands

**Bernd Kowall**

Germany

**Walter Kremers**

USA

**David Kriebel**

USA

**Judith Kulig**

Canada

**Kalyanaraman Kumaran**

UK

**Karen Kuntz**

USA

**Raymond Kuo**

Taiwan

**Emil Kupek**

Brazil

**Oliver Kuss**

Germany

**Lucie Laflamme**

Sweden

**Peter Lane**

UK

**Camille Lassale**

UK

**Nicholas Latimer**

UK

**Anders Ledberg**

Sweden

**Chi Hyun Lee**

USA

**Pak Kuen Philip Lee**

Hong Kong

**Sunghee Lee**

USA

**Mariska M. G. Leeflang**

Netherlands

**Catherine Legrand**

Belgium

**Sam Lendle**

USA

**Liang Li**

USA

**Ruosha Li**

USA

**Dan Liao**

USA

**Lawrence Lin**

USA

**Feng-Chang Lin**

USA

**Melissa Lindeman**

Australia

**Marie Linder**

Sweden

**Aiyi Liu**

USA

**Jordan Louviere**

Australia

**Thomas Love**

USA

**Guobing Lu**

UK

**Qing Lu**

USA

**Jay Lubin**

USA

**Sonja Lumme**

Finland

**Nan Luo**

Singapore

**Sheng Luo**

USA

**Theodore Lystig**

USA

**Joy Macdermid**

Canada

**Andrew Mackinnon**

Australia

**Laura Macpherson**

USA

**Susan Magasi**

USA

**Anselm Mak**

Singapore

**Donald Malec**

USA

**Mario Malicki**

Croatia

**Judith Malmgren**

USA

**Ian Marschner**

Australia

**Dimitris Mavridis**

Greece

**Nancy Mayo**

Canada

**Jim Mccambridge**

UK

**Elaine Mccoll**

UK

**Richard Mceachin**

USA

**Lorcan Mcgarvey**

UK

**Kevin Mcgeechan**

Australia

**David Mclernon**

UK

**Timothy Mcmurry**

USA

**Florian Meinfelder**

Germany

**Joris Menten**

Belgium

**Lisa Merry**

Canada

**Susan Michie**

UK

**Mark Mikkelsen**

USA

**Andrew Miles**

UK

**Peter Molenaar**

USA

**David Morgan**

USA

**Megan Morris**

USA

**Tim Morris**

UK

**Richard Moser**

USA

**Deborah Mullen**

USA

**Katharina Müller**

Germany

**Zachary Munn**

Australia

**Maureen Murdoch**

USA

**Saman Muthukumarana**

Canada

**Duncan Neuhauser**

USA

**Hannelore Neuhauser**

Germany

**Diana Ngo**

USA

**Pentti Nieminen**

Finland

**Theo Niyonsenga**

Australia

**Francis Obare**

Kenya

**Toshiyuki Ojima**

Japan

**Roberto S. Oliveri**

Denmark

**Rumana Omar**

UK

**Igho Onakpoya**

UK

**Dermot O'Reilly**

UK

**Matthew Page**

Australia

**Qing Pan**

USA

**Nikolaos Pandis**

Switzerland

**Xavier Paoletti**

France

**Spyridon Papageorgiou**

Germany

**María Carmen Pardo**

Spain

**Richard Parker**

UK

**Lianne Parkin**

New Zealand

**Eunice Park-Lee**

USA

**Nick Parsons**

UK

**Janet Parsons**

Canada

**Lisa Pastore**

USA

**Sujata Patil**

USA

**Petros Pechlivanoglou**

Canada

**Michael Pencina**

USA

**Lisa Pennells**

UK

**Hans-Peter Piepho**

Germany

**Carol Pierannunzi**

USA

**Sabrina Pit**

Australia

**Maja Pohar Perme**

Slovenia

**Gregory Pond**

Canada

**Diana Porro-Munoz**

Italy

**Corinna Porteri**

Italy

**George Potamias**

Greece

**Matthew Powney**

UK

**Justin Presseau**

UK

**David Prieto-Merino**

UK

**Paolo Emilio Puddu**

Italy

**Guoyou Qin**

China

**Tielin Qin**

USA

**Hude Quan**

Canada

**Gillian Raab**

UK

**Tamara Rader**

Canada

**Shafiqur Rahman**

UK

**Angelique Ralph**

Australia

**Geraldine Rauch**

Germany

**David Reeves**

UK

**Joahnnes Reitsma**

Netherlands

**Matthew Ridd**

UK

**Suzan Robroek**

Netherlands

**José Manuel Rodriguez Barrios**

Spain

**Kris Rogers**

Australia

**Patricia Rogers**

Australia

**Aldo Rosano**

Italy

**Gerta Rücker**

Germany

**Lila Rutten**

USA

**Euijung Ryu**

USA

**Shubhayu Saha**

USA

**Tolulope Sajobi**

Canada

**Joseph Sakshaug**

Germany

**Margaret Sampson**

Canada

**Nancy Santesso**

Canada

**Jonathan Schildcrout**

USA

**Christopher Schmid**

USA

**Rainer Schnell**

Germany

**Shaun Scholes**

UK

**Ellen Schultz**

USA

**Heinzpeter Schwermer**

Switzerland

**Frederic Selck**

USA

**Stephen Senn**

Luxembourg

**Joseph Sewell Araya**

Costa Rica

**Mike Sharland**

UK

**Paul Shekelle**

USA

**Tsung-Jen Shen**

Taiwan

**Irina Shevtsova**

Russian Federation

**Mohamed Shoukri**

Saudi Arabia

**Juned Siddique**

USA

**Becky Skidmore**

Canada

**Rob Smith**

USA

**David Smith**

USA

**Mitchell Smith**

Australia

**Niels Smits**

Netherlands

**Kym Snell**

UK

**Harriet Sommer**

Germany

**Fujian Song**

UK

**Maria Alice Souza De Oliveira Dode**

Brazil

**Bart Spiessens**

Belgium

**Cristian Spitoni**

Netherlands

**James Stamey**

USA

**Renee Stark**

Germany

**Ewout Steyerberg**

Netherlands

**Manfred Stommel**

USA

**Hong Sun**

China

**Joshua Swift**

USA

**Nian-Sheng Tang**

China

**Ge Tao**

USA

**Parisa Tehranifar**

USA

**Armando Teixeira-Pinto**

Australia

**Lucy Thairu**

USA

**James Thomas**

UK

**Daniel Thorburn**

Sweden

**Kristian Thorlund**

Canada

**Andrea Troxel**

USA

**Apostolos Tsiachristas**

Netherlands

**Erick Turner**

USA

**Werner Vach**

Germany

**Linda Valeri**

USA

**Stef Van Buuren**

Netherlands

**Ben Van Calster**

Belgium

**Johanna Van Der Bom**

Netherlands

**Danielle Van Der Windt**

UK

**Emily Van Meter**

USA

**Gert Van Valkenhoef**

Netherlands

**Vassilis Vasdekis**

Greece

**Monica Vasquez**

USA

**Jan Verbakel**

Belgium

**Areti Angeliki Veroniki**

Canada

**Georgia Verropoulou**

Greece

**Andrew Vickers**

USA

**Shankar Viswanathan**

USA

**Monique Margaretha Jozefa Walenkamp**

Netherlands

**Muhammad Walji**

USA

**Stephen John Walters**

UK

**Pengyuan Wang**

USA

**Ming Wang**

USA

**Sijian Wang**

USA

**Mark Weiner**

USA

**Edie Weller**

USA

**Shih-Feng Weng**

Taiwan

**David Wheeler**

USA

**Evelyn Whitlock**

USA

**Marie Wiberg**

Sweden

**Andreas Wienke**

Germany

**Andrew Williams**

USA

**Don Willison**

Canada

**Ed Wilson**

UK

**Carlos King Ho Wong**

Hong Kong

**Alex Wong**

USA

**Lesley Wood**

UK

**Beth Woods**

UK

**Abera Wouhib**

USA

**Kath Wright**

UK

**Changchun Xie**

USA

**Tu Xu**

USA

**Pengcheng Xun**

USA

**Kirsten Yaffe**

USA

**Hsin-Chou Yang**

Taiwan

**Jichen Yang**

USA

**Min Yang**

USA

**Jun Yang**

China

**Wen Ye**

USA

**Zhiying You**

USA

**Tracey Young**

UK

**Recai Yucel**

USA

**Antonia Zapf**

Germany

**Alan Zaslavsky**

USA

**Nikolajs Zeps**

Australia

**Han Zhang**

USA

**Xu Zhang**

USA

**Guangyu Zhang**

USA

**Yang Zhao**

USA

**Wenle Zhao**

USA

**Jin Zhou**

USA

**Muhan Zhou**

USA

**Yijie Zhou**

USA

**Ying Zhou**

USA

**Jihao Zhou**

USA

**Ming Zhu**

USA

